# Septic Arthritis of the Sternoclavicular Joint Manifesting as Sternocleidomastoid Muscle Abscess: A Case Report

**DOI:** 10.7759/cureus.85901

**Published:** 2025-06-13

**Authors:** Linger Sim, Najla Raihan Md Zain, Nurul Syafiqah Norzilan, Khairudin Abdullah

**Affiliations:** 1 Department of Otorhinolaryngology, Kemaman Hospital, Chukai, MYS

**Keywords:** neck abscess, scm abscess, septic arthritis, sternoclavicular joint, sternocleidomastoid muscle

## Abstract

Septic arthritis of the sternoclavicular joint is a rare infection with an insidious onset. It often presents as chest wall swelling. Despite its proximity to vital thoracic and cervical structures, extension of infection beyond the sternoclavicular joint is uncommon. Among the rare complications, the development of a neck abscess is extremely unusual. Awareness of such atypical presentation should be emphasized. We present a case of sternocleidomastoid muscle abscess that was preceded by septic arthritis of the sternoclavicular joint in an elderly patient with diabetes.

## Introduction

The sternoclavicular joint is a diarthrodial joint located between the manubrium and clavicle. Septic arthritis of the sternoclavicular joint is uncommon, accounting for only 1% of all bone and joint infections. Risk factors include an immunocompromised state, intravenous drug use, central venous catheter placement, trauma, rheumatoid arthritis, and malignancy [1]. The most common pathogen responsible for septic arthritis is *Staphylococcus aureus,* while *Streptococcus pneumoniae* and *Streptococcus pyogenes* are also commonly involved. Patients may present with fever, joint swelling, warmth, and reduced mobility. It is rarely diagnosed at the first visit, as the initial symptoms are often mild and subtle. Delayed diagnosis can lead to severe complications such as abscess, mediastinitis, osteomyelitis, septic shock, and superior vena cava syndrome [2]. Neck abscesses as a complication of septic arthritis in the sternoclavicular joint are extremely rare, with only four cases reported in the English literature [3-5]. Among these, only one case presented as an abscess of the sternocleidomastoid (SCM) muscle [5]. Here, we report a case of SCM abscess resulting from septic arthritis of the sternoclavicular joint and discuss the surgical management. 

## Case presentation

A 60-year-old man with diabetes mellitus presented to the emergency department with a complaint of painful right neck swelling for three weeks. The swelling started in the anterior chest region and progressively increased to the right lower neck region, causing reduced mobility in the neck and right shoulder. It was associated with fever, lethargy, and dysphagia. Other than diabetes mellitus, he had no additional risk factors such as prior trauma, central line use, intravenous drug use, or infection at a distant site. He was prescribed a two-week course of oral antibiotics by a general practitioner, but the symptoms did not improve.

**Figure 1:**
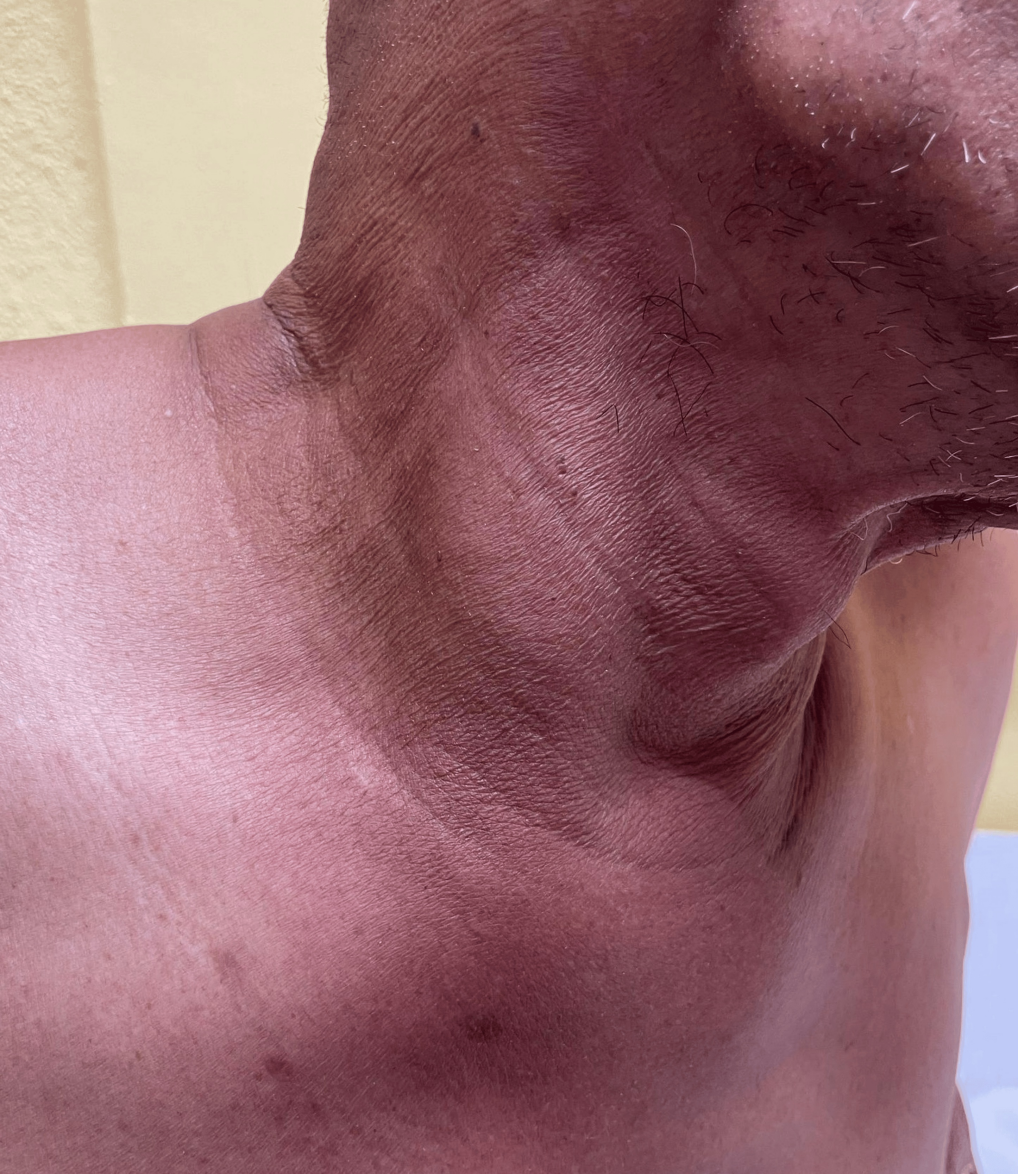
Right neck swelling at level III and IV, along with diffuse swelling on the right upper chest over the medial end of right clavicle

Examination showed a tender and firm right neck swelling at level III and IV, along with diffuse swelling on the right upper chest over the medial end of the right clavicle (Figure 1). There was a reduced range of motion in the right shoulder. Laboratory investigations showed leukocytosis, with a white blood cell count of 12 × 10⁹/L, and hyperglycemia, with a blood glucose level of 20 mmol/L. Differential diagnoses included SCM abscess, suppurative lymphadenitis, and deep neck space infection.

**Figure 2:**
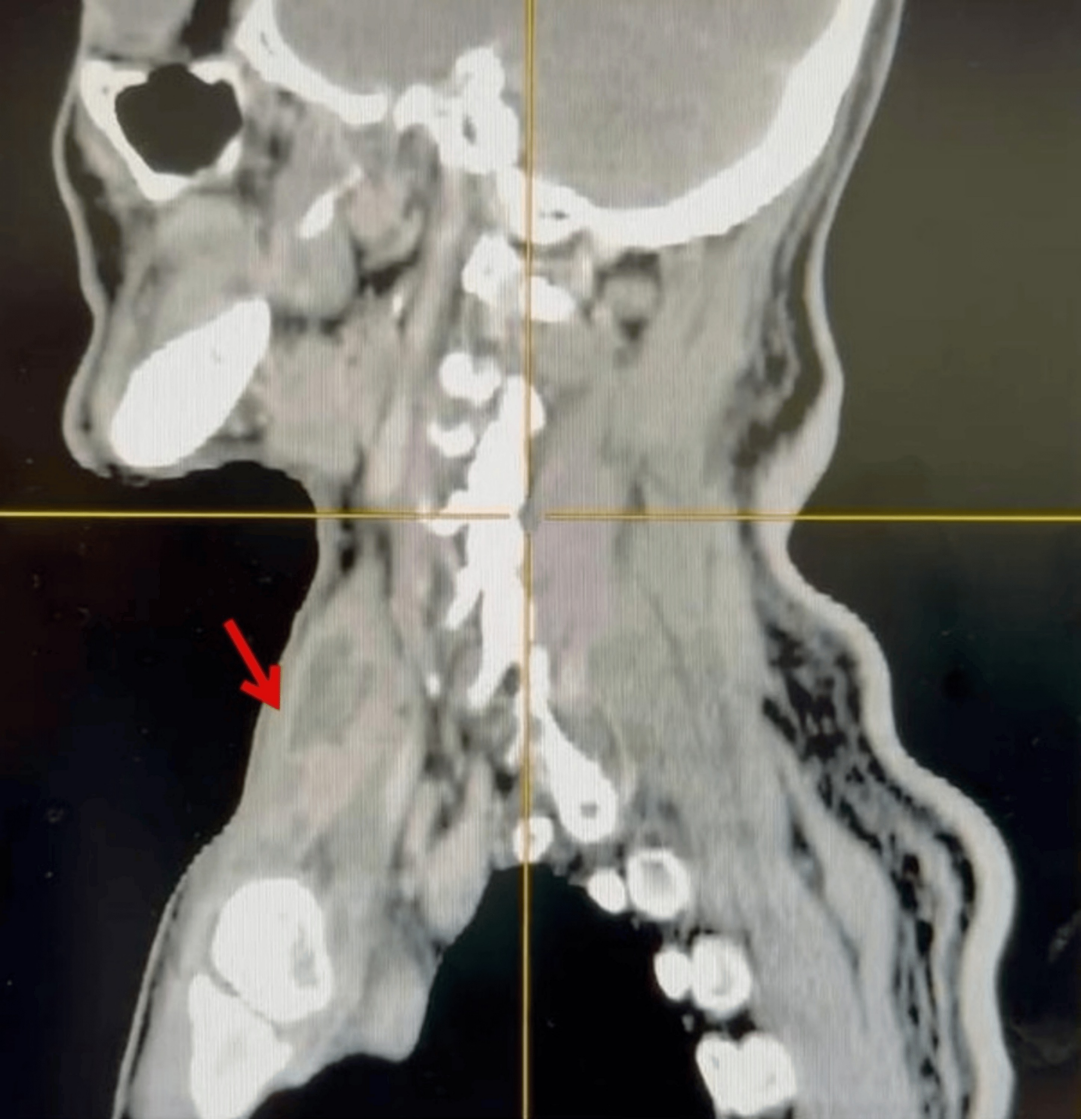
Sagittal view of CECT neck showing collection within the inferior right SCM muscle CECT: contrast-enhanced computed tomography; SCM: sternocleidomastoid

**Figure 3:**
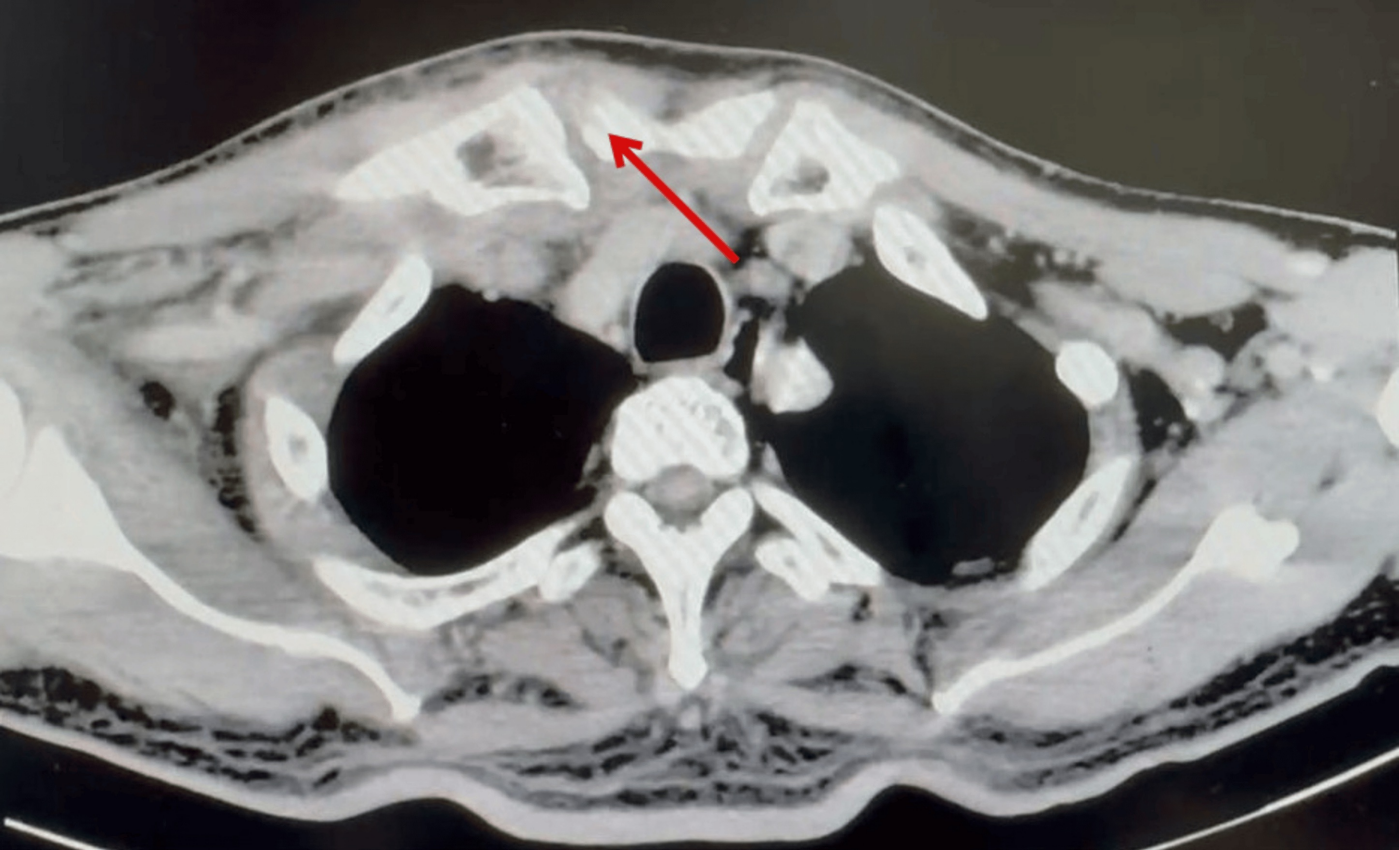
Erosion of right sternoclavicular joint

Contrast-enhanced computed tomography (CECT) of the neck revealed a multiloculated, rim-enhancing hypodense collection with air pockets within the inferior right SCM muscle, measuring 2 x 1 x 4 cm, extending from the level of C4 to its distal insertion at the medial end of the right clavicle (Figure 2), confirming the diagnosis of an SCM abscess. Erosion of the right sternoclavicular joint and air pockets at the medial end of the right clavicle were suggestive of osteomyelitic changes (Figure 3). Additionally, a filling defect in the right brachiocephalic vein was consistent with thrombosis.

**Figure 4:**
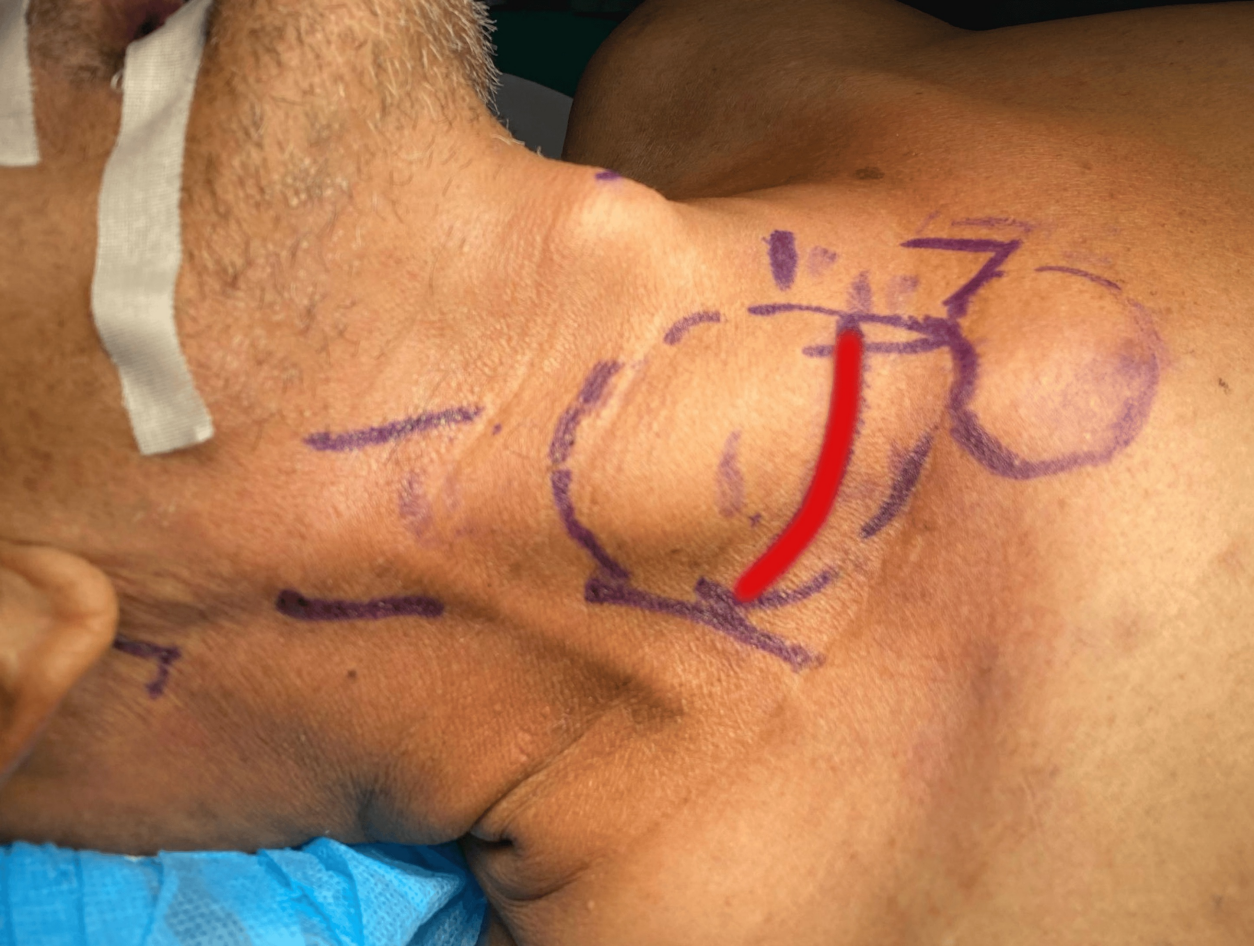
Surface marking for incision and drainage, following the skin crease over the swelling, two finger-breadths above the clavicle (red line: incision site)

**Figure 5:**
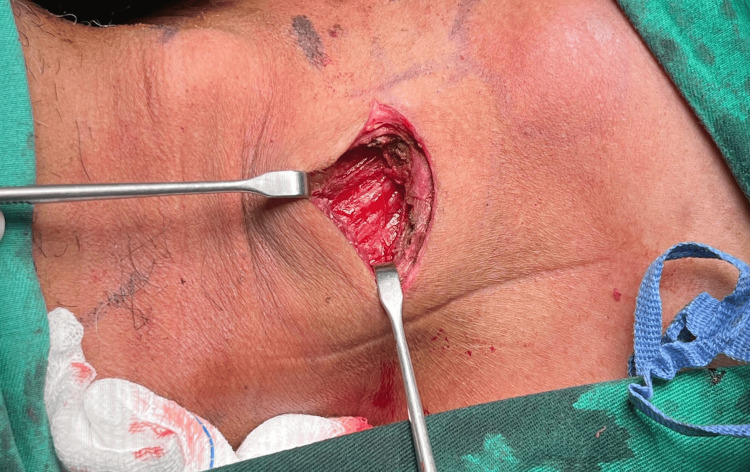
Abscess cavity within right SCM muscle extending from the level of hyoid down to the medial end of the right clavicle SCM: sternocleidomastoid

The patient subsequently underwent incision and drainage of the abscess, with the incision made following the skin crease over the swelling, two finger-breadths above the clavicle (Figure 4). The incision was chosen because it was located at the dependent lower end of the SCM, which allowed effective drainage and provided access for dissection to the medial end of the clavicle. Intraoperatively, 5 cc of pus was drained from the right SCM. Blunt dissection of the locules was performed, with the cavity extending from the level of the hyoid down to the medial end of the right clavicle (Figure 5). 

Cultures showed no growth, likely due to prior partial treatment with oral antibiotics. The patient showed clinical improvement after one week of intravenous ampicillin-sulbactam and was then discharged on oral ampicillin-sulbactam for an additional four weeks, following improvement in inflammatory markers. At discharge, the C-reactive protein (CRP) level had decreased to 0.55 mg/L, and the erythrocyte sedimentation rate (ESR) was 30 mm/hour. The antibiotic was chosen because it is a broad-spectrum agent with coverage against *S. aureus*. No recurrence was observed throughout the three-month follow-up.

## Discussion

Given the close proximity of the sternoclavicular joint to the vital structures such as the mediastinum, pleural cavity, and brachiocephalic vessels, it is important to recognize the clinical signs of an infected joint. As demonstrated in this case, suspicion should be raised when the patient reports that the swelling began in the anterior chest region and he was unable to lift his shoulder. During normal shoulder motion, the sternoclavicular joint contributes 30-35° of elevation, a similar range of motion in the horizontal plane, and 45° of rotation along its long axis [6].

In this case, the formation of the SCM abscess was believed to result from direct extension of the infection from the sternoclavicular joint through the sternal head insertion of the muscle. As the infection progressed, erosion of the joint capsule and adjacent bony cortex (sternum and clavicle) occurred, allowing the infectious process to invade the overlying SCM muscle. When reviewing the scan, it is essential to evaluate the sternoclavicular joint to ensure septic arthritis is not overlooked. The same applies to the nearby vital structures, such as the presence of brachiocephalic vein thrombosis in this patient.

Empiric antibiotic therapy should cover *S. aureus*, typically with agents like oxacillin or cefazolin. However, if the patient has risk factors for methicillin-resistant *S. aureus* (MRSA) such as intravenous drug use, hemodialysis, central venous catheterization, recent hospitalization, or if MRSA prevalence is high in the community, treatment should be initiated with vancomycin or clindamycin instead. Antibiotic therapy can be adjusted based on culture results from aspirates or samples obtained during incision and drainage. A treatment duration of around four weeks is usually advised for septic arthritis, with an extension to six weeks if osteomyelitis is also present, which is common in sternoclavicular joint infections [7,8].

In a review of 180 cases of sternoclavicular septic arthritis, surgery was performed in 58% of patients [8]. Surgical interventions ranged from limited debridement of necrotic bone and soft tissue to extensive procedures including en-bloc resection of the sternoclavicular joint. Eleven patients who were initially managed medically experienced treatment failure and subsequently required surgery after 6-90 days of antibiotic therapy. The average duration of antibiotics was 41 days for patients treated non-surgically and 52 days for those who underwent surgery, likely reflecting a higher incidence of complications such as osteomyelitis and mediastinitis in the surgical group.

## Conclusions

This case highlights a rare complication of septic arthritis of the sternoclavicular joint, which should be considered in the differential diagnosis of lower neck abscesses, particularly in immunocompromised individuals. Early imaging, especially CECT neck, is recommended in high-risk patients not only to localize the abscess or collection but also to identify uncommon sources of infection, such as the sternoclavicular joint.
